# Poly[[{μ_3_-dihydrogen [(pyridin-4-yl­methyl­imino)­bis­(methyl­ene)]diphos­phon­ato-κ^5^ *O*:*O*′,*N*,*O*′′:*N*′}copper(II)] dihydrate]

**DOI:** 10.1107/S1600536811052512

**Published:** 2011-12-10

**Authors:** Shi-Yong Zhang, Zhong-Gao Zhou, Ke-Jun Wang

**Affiliations:** a College of Chemistry and Chemical Engineering, Gannan Normal University, Ganzhou, Jiangxi 341000, People’s Republic of China

## Abstract

In the title polymer, {[Cu(C_8_H_12_N_2_O_6_P_2_)]·2H_2_O}_*n*_, the geometry of the five-coordinate Cu^II^ ion can best be described as slightly distorted square-pyramidal formed by one N and two O atoms of an N(CH_2_PO_3_H)_2_ group and one N atom from a pyridine ring. The elongated apex of the pyramid is occupied by one O atom from a third diphospho­nate ligand. The inter­connection of Cu^2+^ ions by the diphospho­nate ligands results in the formation of a double-chain array along the *b* axis, in which the two sub-chains are inter­locked by pairs of PO_3_ groups. The outside of each sub-chain is decorated by other PO_3_ groups. These double chains are further assembled into a three-dimensional supra­molecular architecture *via* a large number of O—H⋯O hydrogen bonds between the phospho­nate groups and lattice water mol­ecules.

## Related literature

For background to metal phospho­nate chemistry, see: Maeda (2004 ▶); Mao (2007 ▶); Shimizu *et al.* (2009 ▶). For the synthetic strategy of attaching functional groups to a phospho­nic acid ligand, see: Drumel *et al.* (1995 ▶); Mao *et al.* (2002 ▶); Liang & Shimizu (2007 ▶); Du *et al.* (2006 ▶, 2010*b* ▶). For a structurally related complex, see: Song & Mao (2005 ▶). For the zwitterionic behavior of amino­phospho­nic acid, see: Yang *et al.* (2008 ▶); Du *et al.* (2009 ▶, 2010*a* ▶).
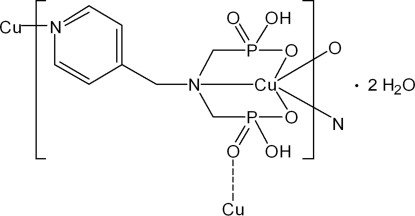


## Experimental

### 

#### Crystal data

[Cu(C_8_H_12_N_2_O_6_P_2_)]·2H_2_O
*M* *_r_* = 393.71
Triclinic, 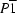

*a* = 8.9250 (3) Å
*b* = 9.0000 (3) Å
*c* = 10.5066 (3) Å
α = 75.648 (2)°
β = 67.124 (2)°
γ = 67.126 (2)°
*V* = 711.75 (4) Å^3^
*Z* = 2
Mo *K*α radiation
μ = 1.80 mm^−1^
*T* = 296 K
0.40 × 0.03 × 0.02 mm

#### Data collection

Bruker APEXII CCD diffractometer
Absorption correction: multi-scan (*SADABS*; Bruker, 2008 ▶) *T*_min_ = 0.605, *T*_max_ = 0.746
7659 measured reflections
3267 independent reflections
2309 reflections with *I* > 2σ(*I*)
*R*_int_ = 0.043

#### Refinement

*R*[*F* ^2^ > 2σ(*F* ^2^)] = 0.046
*wR*(*F* ^2^) = 0.118
*S* = 1.03
3267 reflections
202 parameters
6 restraints
H atoms treated by a mixture of independent and constrained refinement
Δρ_max_ = 0.50 e Å^−3^
Δρ_min_ = −0.60 e Å^−3^

### 

Data collection: *APEX2* (Bruker, 2008 ▶); cell refinement: *SAINT* (Bruker, 2008 ▶); data reduction: *SAINT*; program(s) used to solve structure: *SHELXS97* (Sheldrick, 2008 ▶); program(s) used to refine structure: *SHELXL97* (Sheldrick, 2008 ▶); molecular graphics: *SHELXTL* (Sheldrick, 2008 ▶) and *DIAMOND* (Brandenburg, 2010 ▶); software used to prepare material for publication: *SHELXTL*.

## Supplementary Material

Crystal structure: contains datablock(s) I, global. DOI: 10.1107/S1600536811052512/fj2483sup1.cif

Structure factors: contains datablock(s) I. DOI: 10.1107/S1600536811052512/fj2483Isup2.hkl

Additional supplementary materials:  crystallographic information; 3D view; checkCIF report

## Figures and Tables

**Table 1:** Hydrogen-bond geometry (Å, °)

*D*—H⋯*A*	*D*—H	H⋯*A*	*D*⋯*A*	*D*—H⋯*A*
O1—H1*B*⋯O1*W*	0.82	1.68	2.494 (4)	169
O6—H6*C*⋯O2*W*	0.82	1.75	2.567 (5)	172
O1*W*—H1*WA*⋯O5^i^	0.83 (2)	1.92 (2)	2.746 (4)	177 (5)
O1*W*—H1*WB*⋯O5^ii^	0.84 (2)	1.93 (2)	2.747 (4)	167 (5)
O2*W*—H2*WA*⋯O1^iii^	0.85 (2)	2.09 (3)	2.882 (4)	155 (5)
O2*W*—H2*WB*⋯O3^iv^	0.85 (2)	1.96 (3)	2.776 (4)	161 (6)

Symmetry codes: (i) 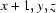; (ii) 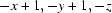; (iii) 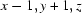; (iv) 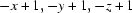.

**Fig. 1.**
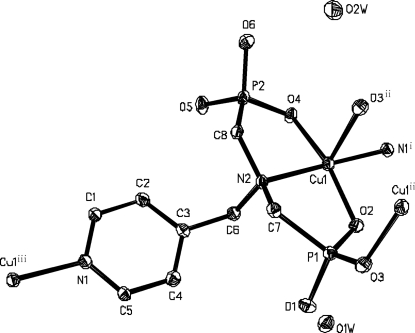
View of the selected unit of (I), showing the atom-numbering scheme. Displacement ellipsoids are drawn at the 30% probability level. [Symmetry codes: (i) x, y + 1, z; (ii) -x + 1, -y + 1, -z + 1; (iii) x, y - 1, z.]

**Fig. 2.**
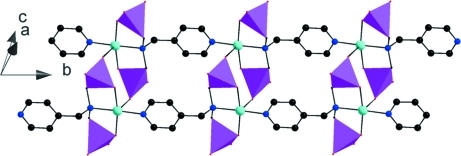
View of the double-chain structure of (I) along the b-axis. The CPO3 tetrahedra are shaded in purple. Cu, N and C atoms are drawn as cyan, blue and black circles, respectively.

**Fig. 3.**
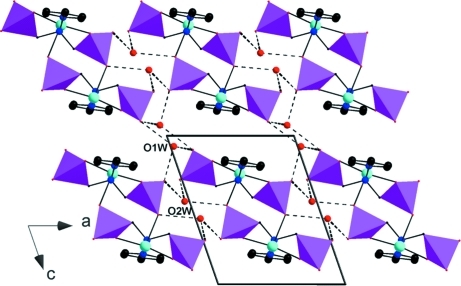
View of the structure of (I) down the b-axis. For display details, see the caption for Fig. 2. Hydrogen bonds are represented by dashed lines.

## supplementary crystallographic information

### Comment

During the past few decades, the syntheses of metal phosphonates with various structures has attracted much attention, owing to their potential applications in areas such as catalysis, ion exchange, intercalation chemistry, and material chemistry (Maeda, 2004; Mao, 2007; Shimizu *et al.*, 2009). The strategy of attaching functional groups such as amine, hydroxyl, carboxylate, sulfonate, and sulfone groups to the phosphonic acid has proven to be an effective method for the isolation of a variety of metal phosphonates with new structures (Drumel *et al.*, 1995; Mao *et al.*, 2002; Liang & Shimizu, 2007; Du *et al.* 2006, 2010*b*). Recently, we are interested in the combination of multiple functional groups to phosphonic acid as a more complex ligand. Herein, we report a copper(II) phosphonate based on an amino-bis(methyl-phosphonic acid) ligand, which contains pyridyl group as an additional functional group. As far as we are aware, only one layered cobalt(II) phosphonate has been reported based on the same ligand (Song & Mao, 2005).

The title compound (I) features a one-dimensional double-chain structure. The formula of it contains one Cu^2+^ ion, one H_2_*L*^2-^ anion and two lattice water molecules. Cu(1) ion is five-coordinate and its coordination geometry can be described as a slightly distorted square-pyramid (Fig. 1): the square plane is formed by one N and two O atoms of a N(CH_2_PO_3_H)_2_ group as well as one N atom of a pyridyl group from two H_2_*L*^2-^ ligand, and the prolonged apex of the pyramid is occupied by one O atom from a third H_2_*L*^2-^ ligand. The H_2_*L*^2-^ ligand in compound (I) acts as a pentadentate chelating and bridging ligand. It chelates one Cu^2+^ ion by its N(CH_2_PO_3_H)_2_ group in a tridentate fashion (2O and 1 N), and also bridges with other two Cu^2+^ ions *via* its pyridyl group and a third O atom (Scheme 1). The two phosphonate groups of the H_2_*L*^2-^ ligand both are 1*H*-protonated as the requirement for charge balance and also as indicated by two much longer P—O bonds. It is worthy of note that the strongly basic N atom in the H_2_*L*^2-^ ligand is not protonated but bonded to a Cu^2+^ ion, which is rarely observed for phosphonic acid ligands containing a tertiary amine group (Yang *et al.*, 2008; Du *et al.*, 2009, 2010*a*).

The interconnection of the Cu^2+^ ions by the H*L*^2-^ anions results in the formation of a one-dimensional double-chain array along the *b*-axis, in which the two sub-chains are inter-locked by pairs of P(1)O_3_ groups and the outside of each sub-chain is decorated by P(2)O_3_ groups. It is worthy of note that such two sub-chains are related by inversion centers, and the shortest Cd···Cd distance between them is 5.170 (4) Å while that in each sub-chain is 9.000 (1) Å. These double-chains are further assembled into a three-dimensional supramolecular architecture *via* a large number of hydrogen bonds between the phosphonate groups and lattice water molecules (Fig. 3 and Table 1).

### Experimental

4-Pyridyl-CH_2_N(CH_2_PO_3_H_2_)_2_ (0.2 mmol) was dissolved in 4 ml H_2_O and poured into a test tube, then CuCl_2_.2H_2_O (0.15 mmol) dissolved in 8 ml EtOH was carefully layered onto it and left to stand at room temperature. Blue column-shaped crystals of (I) were obtained after about two weeks later. IR data for (I) (KBr, cm^-1^): 3477(*s*), 3228(*s*), 3141(*m*), 3079(*m*), 2975(*m*), 2913(*m*), 2376(*m*), 1846(*m*), 1624(*s*), 1502(*m*), 1448(*m*), 1433(*m*), 1350(*m*), 1266(*s*), 1221(*m*), 1173(*s*), 1140(*versus*), 1065(*s*), 1051(*s*), 1032(*s*), 945(*s*), 929(*m*), 906(*m*), 866(*m*), 844(*m*), 795(*m*), 742(*m*), 648(*m*), 588(*s*), 524(*m*), 482(*m*), 453(*m*).

### Refinement

C-bound H atoms were placed in idealized positions and constrained to ride on their parent atoms, with C—H distances of 0.93 or 0.97 Å, and with *U*_iso_(H) = 1.2*U*_eq_(C). H atoms of –PO_3_H^-^ groups were also placed in idealized positions and constrained to ride on their parent atoms, with O—H distances of 0.82 Å, and with *U*_iso_(H) = 1.2*U*_eq_(O). Water H atoms were located in a difference map and refined with *U*_iso_(H) values set at 1.5*U*_eq_(O). The O—H distances of water were restrained to be 0.85 (1) Å.

### Crystal data

**Table:**

[Cu(C_8_H_12_N_2_O_6_P_2_)]·2H_2_O	*Z* = 2
*M**_r_* = 393.71	*F*(000) = 402
Triclinic, *P*1	*D*_x_ = 1.837 Mg m^−^^3^
Hall symbol: -P 1	Mo *K*α radiation, λ = 0.71073 Å
*a* = 8.9250 (3) Å	Cell parameters from 1430 reflections
*b* = 9.0000 (3) Å	θ = 2.1–27.6°
*c* = 10.5066 (3) Å	µ = 1.80 mm^−^^1^
α = 75.648 (2)°	*T* = 296 K
β = 67.124 (2)°	Needle, blue
γ = 67.126 (2)°	0.40 × 0.03 × 0.02 mm
*V* = 711.75 (4) Å^3^	

### Data collection

**Table:**

Bruker APEXII CCD diffractometer	3267 independent reflections
Radiation source: fine-focus sealed tube	2309 reflections with *I* > 2σ(*I*)
graphite	*R*_int_ = 0.043
phi and ω scans	θ_max_ = 27.6°, θ_min_ = 2.1°
Absorption correction: multi-scan (*SADABS*; Bruker, 2008)	*h* = −10→11
*T*_min_ = 0.605, *T*_max_ = 0.746	*k* = −11→11
7659 measured reflections	*l* = −13→13

### Refinement

**Table:**

Refinement on *F*^2^	Primary atom site location: structure-invariant direct methods
Least-squares matrix: full	Secondary atom site location: difference Fourier map
*R*[*F*^2^ > 2σ(*F*^2^)] = 0.046	Hydrogen site location: inferred from neighbouring sites
*wR*(*F*^2^) = 0.118	H atoms treated by a mixture of independent and constrained refinement
*S* = 1.03	*w* = 1/[σ^2^(*F*_o_^2^) + (0.055*P*)^2^ + 0.1702*P*] where *P* = (*F*_o_^2^ + 2*F*_c_^2^)/3
3267 reflections	(Δ/σ)_max_ < 0.001
202 parameters	Δρ_max_ = 0.50 e Å^−^^3^
6 restraints	Δρ_min_ = −0.60 e Å^−^^3^

### Special details

**Table:**

Geometry. All e.s.d.'s (except the e.s.d. in the dihedral angle between two l.s. planes) are estimated using the full covariance matrix. The cell e.s.d.'s are taken into account individually in the estimation of e.s.d.'s in distances, angles and torsion angles; correlations between e.s.d.'s in cell parameters are only used when they are defined by crystal symmetry. An approximate (isotropic) treatment of cell e.s.d.'s is used for estimating e.s.d.'s involving l.s. planes.
Refinement. Refinement of *F*^2^ against ALL reflections. The weighted *R*-factor *wR* and goodness of fit *S* are based on *F*^2^, conventional *R*-factors *R* are based on *F*, with *F* set to zero for negative *F*^2^. The threshold expression of *F*^2^ > σ(*F*^2^) is used only for calculating *R*-factors(gt) *etc*. and is not relevant to the choice of reflections for refinement. *R*-factors based on *F*^2^ are statistically about twice as large as those based on *F*, and *R*- factors based on ALL data will be even larger.

### Fractional atomic coordinates and isotropic or equivalent isotropic displacement parameters (Å^2^)

**Table:**

	*x*	*y*	*z*	*U*_iso_*/*U*_eq_	
Cu1	0.47006 (6)	0.60143 (5)	0.25556 (5)	0.02485 (17)	
P1	0.69833 (14)	0.32696 (12)	0.39341 (11)	0.0237 (2)	
P2	0.17163 (14)	0.57368 (13)	0.21239 (12)	0.0265 (3)	
N1	0.5076 (4)	−0.1825 (4)	0.2025 (3)	0.0240 (7)	
N2	0.4396 (4)	0.3746 (4)	0.2980 (3)	0.0207 (7)	
C1	0.3823 (5)	−0.0485 (5)	0.1777 (4)	0.0282 (9)	
H1A	0.2869	−0.0590	0.1696	0.034*	
C2	0.3912 (5)	0.1042 (5)	0.1641 (4)	0.0293 (10)	
H2A	0.3025	0.1945	0.1457	0.035*	
C3	0.5301 (5)	0.1261 (4)	0.1773 (4)	0.0233 (9)	
C4	0.6596 (6)	−0.0148 (5)	0.1992 (4)	0.0294 (9)	
H4A	0.7570	−0.0076	0.2065	0.035*	
C5	0.6465 (5)	−0.1652 (5)	0.2102 (4)	0.0273 (9)	
H5A	0.7364	−0.2575	0.2233	0.033*	
C6	0.5389 (5)	0.2920 (4)	0.1674 (4)	0.0243 (9)	
H6A	0.4964	0.3607	0.0933	0.029*	
H6B	0.6587	0.2831	0.1412	0.029*	
C7	0.5053 (5)	0.2840 (5)	0.4152 (4)	0.0235 (9)	
H7A	0.5324	0.1684	0.4164	0.028*	
H7B	0.4179	0.3168	0.5029	0.028*	
C8	0.2500 (5)	0.4113 (5)	0.3387 (4)	0.0224 (8)	
H8A	0.1931	0.4454	0.4312	0.027*	
H8B	0.2255	0.3151	0.3391	0.027*	
O1	0.8567 (4)	0.1971 (3)	0.3047 (3)	0.0309 (7)	
H1B	0.9034	0.2423	0.2302	0.046*	
O2	0.6750 (3)	0.4935 (3)	0.3111 (3)	0.0278 (6)	
O3	0.7175 (4)	0.3101 (3)	0.5319 (3)	0.0307 (7)	
O4	0.2907 (3)	0.6731 (3)	0.1706 (3)	0.0273 (6)	
O5	0.1664 (4)	0.5097 (3)	0.0961 (3)	0.0356 (7)	
O6	−0.0163 (4)	0.6712 (4)	0.2955 (3)	0.0393 (8)	
H6C	−0.0137	0.7334	0.3394	0.059*	
O1W	1.0256 (4)	0.3000 (4)	0.0716 (3)	0.0381 (8)	
H1WA	1.065 (6)	0.366 (5)	0.078 (5)	0.057*	
H1WB	0.964 (6)	0.344 (5)	0.021 (5)	0.057*	
O2W	−0.0383 (5)	0.8732 (4)	0.4421 (4)	0.0462 (9)	
H2WA	−0.037 (7)	0.965 (4)	0.397 (5)	0.069*	
H2WB	0.053 (5)	0.832 (6)	0.464 (6)	0.069*	

### Atomic displacement parameters (Å^2^)

**Table:**

	*U*^11^	*U*^22^	*U*^33^	*U*^12^	*U*^13^	*U*^23^
Cu1	0.0279 (3)	0.0187 (3)	0.0339 (3)	−0.0064 (2)	−0.0186 (2)	−0.0011 (2)
P1	0.0258 (6)	0.0185 (5)	0.0290 (6)	−0.0027 (4)	−0.0154 (5)	−0.0030 (4)
P2	0.0283 (6)	0.0233 (5)	0.0369 (6)	−0.0077 (5)	−0.0215 (5)	−0.0020 (5)
N1	0.0277 (18)	0.0195 (17)	0.0268 (19)	−0.0072 (14)	−0.0117 (15)	−0.0025 (14)
N2	0.0248 (17)	0.0177 (16)	0.0235 (17)	−0.0068 (14)	−0.0127 (15)	−0.0014 (13)
C1	0.026 (2)	0.023 (2)	0.042 (3)	−0.0039 (17)	−0.018 (2)	−0.0081 (19)
C2	0.030 (2)	0.022 (2)	0.040 (3)	−0.0025 (18)	−0.020 (2)	−0.0054 (18)
C3	0.028 (2)	0.0192 (19)	0.023 (2)	−0.0061 (17)	−0.0087 (18)	−0.0038 (16)
C4	0.031 (2)	0.028 (2)	0.034 (2)	−0.0097 (19)	−0.015 (2)	−0.0042 (19)
C5	0.028 (2)	0.0169 (19)	0.039 (3)	−0.0065 (17)	−0.017 (2)	−0.0003 (18)
C6	0.028 (2)	0.022 (2)	0.027 (2)	−0.0063 (17)	−0.0142 (18)	−0.0024 (17)
C7	0.031 (2)	0.0188 (19)	0.023 (2)	−0.0078 (17)	−0.0125 (18)	0.0005 (16)
C8	0.023 (2)	0.022 (2)	0.026 (2)	−0.0100 (17)	−0.0100 (18)	−0.0025 (17)
O1	0.0302 (16)	0.0228 (15)	0.0335 (17)	−0.0006 (13)	−0.0123 (14)	−0.0028 (13)
O2	0.0279 (16)	0.0194 (14)	0.0395 (17)	−0.0054 (12)	−0.0194 (14)	0.0008 (13)
O3	0.0312 (17)	0.0335 (16)	0.0321 (17)	−0.0054 (13)	−0.0200 (14)	−0.0043 (13)
O4	0.0315 (16)	0.0234 (14)	0.0367 (17)	−0.0100 (12)	−0.0243 (14)	0.0038 (13)
O5	0.049 (2)	0.0308 (16)	0.0431 (19)	−0.0148 (15)	−0.0302 (16)	−0.0026 (14)
O6	0.0290 (17)	0.0360 (18)	0.061 (2)	−0.0048 (14)	−0.0248 (16)	−0.0111 (16)
O1W	0.041 (2)	0.0387 (19)	0.042 (2)	−0.0163 (16)	−0.0204 (16)	0.0014 (15)
O2W	0.048 (2)	0.0337 (19)	0.061 (2)	−0.0053 (17)	−0.030 (2)	−0.0057 (17)

### Geometric parameters (Å, °)

**Table:**

Cu1—O2	1.949 (3)	C2—C3	1.390 (5)
Cu1—O4	1.949 (2)	C2—H2A	0.9300
Cu1—N1^i^	2.008 (3)	C3—C4	1.385 (5)
Cu1—N2	2.080 (3)	C3—C6	1.501 (5)
Cu1—O3^ii^	2.315 (3)	C4—C5	1.376 (5)
P1—O3	1.495 (3)	C4—H4A	0.9300
P1—O2	1.514 (3)	C5—H5A	0.9300
P1—O1	1.570 (3)	C6—H6A	0.9700
P1—C7	1.827 (4)	C6—H6B	0.9700
P2—O5	1.497 (3)	C7—H7A	0.9700
P2—O4	1.518 (3)	C7—H7B	0.9700
P2—O6	1.563 (3)	C8—H8A	0.9700
P2—C8	1.831 (4)	C8—H8B	0.9700
N1—C1	1.340 (5)	O1—H1B	0.8200
N1—C5	1.341 (5)	O3—Cu1^ii^	2.315 (3)
N1—Cu1^iii^	2.008 (3)	O6—H6C	0.8200
N2—C7	1.489 (5)	O1W—H1WA	0.832 (19)
N2—C8	1.492 (5)	O1W—H1WB	0.836 (19)
N2—C6	1.507 (5)	O2W—H2WA	0.846 (19)
C1—C2	1.376 (6)	O2W—H2WB	0.848 (19)
C1—H1A	0.9300		
			
O2—Cu1—O4	167.12 (11)	C3—C2—H2A	119.4
O2—Cu1—N1^i^	93.65 (12)	C4—C3—C2	115.6 (3)
O4—Cu1—N1^i^	92.92 (12)	C4—C3—C6	122.4 (4)
O2—Cu1—N2	86.48 (11)	C2—C3—C6	121.9 (3)
O4—Cu1—N2	86.29 (11)	C5—C4—C3	121.1 (4)
N1^i^—Cu1—N2	176.50 (13)	C5—C4—H4A	119.4
O2—Cu1—O3^ii^	96.97 (11)	C3—C4—H4A	119.4
O4—Cu1—O3^ii^	94.37 (11)	N1—C5—C4	121.9 (4)
N1^i^—Cu1—O3^ii^	87.53 (12)	N1—C5—H5A	119.0
N2—Cu1—O3^ii^	95.93 (11)	C4—C5—H5A	119.0
O3—P1—O2	116.53 (16)	C3—C6—N2	115.5 (3)
O3—P1—O1	108.57 (16)	C3—C6—H6A	108.4
O2—P1—O1	110.13 (17)	N2—C6—H6A	108.4
O3—P1—C7	110.01 (18)	C3—C6—H6B	108.4
O2—P1—C7	103.88 (16)	N2—C6—H6B	108.4
O1—P1—C7	107.31 (17)	H6A—C6—H6B	107.5
O5—P2—O4	115.75 (17)	N2—C7—P1	109.1 (3)
O5—P2—O6	108.56 (17)	N2—C7—H7A	109.9
O4—P2—O6	111.15 (16)	P1—C7—H7A	109.9
O5—P2—C8	112.42 (17)	N2—C7—H7B	109.9
O4—P2—C8	103.00 (16)	P1—C7—H7B	109.9
O6—P2—C8	105.44 (18)	H7A—C7—H7B	108.3
C1—N1—C5	118.3 (3)	N2—C8—P2	108.1 (3)
C1—N1—Cu1^iii^	120.0 (3)	N2—C8—H8A	110.1
C5—N1—Cu1^iii^	121.1 (3)	P2—C8—H8A	110.1
C7—N2—C8	111.7 (3)	N2—C8—H8B	110.1
C7—N2—C6	112.3 (3)	P2—C8—H8B	110.1
C8—N2—C6	112.8 (3)	H8A—C8—H8B	108.4
C7—N2—Cu1	107.6 (2)	P1—O1—H1B	109.5
C8—N2—Cu1	104.3 (2)	P1—O2—Cu1	119.08 (16)
C6—N2—Cu1	107.7 (2)	P1—O3—Cu1^ii^	133.88 (16)
N1—C1—C2	121.7 (4)	P2—O4—Cu1	117.93 (16)
N1—C1—H1A	119.1	P2—O6—H6C	109.5
C2—C1—H1A	119.1	H1WA—O1W—H1WB	109 (4)
C1—C2—C3	121.3 (4)	H2WA—O2W—H2WB	107 (4)
C1—C2—H2A	119.4		

Symmetry codes: (i) *x*, *y*+1, *z*; (ii) −*x*+1, −*y*+1, −*z*+1; (iii) *x*, *y*−1, *z*.

### Hydrogen-bond geometry (Å, °)

**Table:**

*D*—H···*A*	*D*—H	H···*A*	*D*···*A*	*D*—H···*A*
O1—H1B···O1W	0.82	1.68	2.494 (4)	169
O6—H6C···O2W	0.82	1.75	2.567 (5)	172
O1W—H1WA···O5^iv^	0.83 (2)	1.92 (2)	2.746 (4)	177 (5)
O1W—H1WB···O5^v^	0.84 (2)	1.93 (2)	2.747 (4)	167 (5)
O2W—H2WA···O1^vi^	0.85 (2)	2.09 (3)	2.882 (4)	155 (5)
O2W—H2WB···O3^ii^	0.85 (2)	1.96 (3)	2.776 (4)	161 (6)

Symmetry codes: (iv) *x*+1, *y*, *z*; (v) −*x*+1, −*y*+1, −*z*; (vi) *x*−1, *y*+1, *z*; (ii) −*x*+1, −*y*+1, −*z*+1.

## References

[bb1] Brandenburg, K. (2010). *DIAMOND.* Crystal Impact GbR, Bonn, Germany.

[bb2] Bruker (2008). *APEX2*, *SADABS* and *SAINT* Bruker AXS Inc., Madison, Wisconsin, USA.

[bb3] Drumel, S., Janvier, P., Deniaud, D. & Bujoli, B. (1995). *Chem. Commun.* pp. 1051–1052.

[bb4] Du, Z.-Y., Sun, Y.-H., Liu, Q.-Y., Xie, Y.-R. & Wen, H.-R. (2009). *Inorg. Chem.* **48**, 7015–7017. 10.1021/ic901130a 19583245

[bb5] Du, Z.-Y., Sun, Y.-H., Zhang, X.-Z., Luo, S.-F., Xie, Y.-R. & Wan, D.-B. (2010*a*). *CrystEngComm*, **12**, 1774–1778.

[bb6] Du, Z.-Y., Wen, H.-R., Liu, C.-M., Sun, Y.-H., Lu, Y.-B. & Xie, Y.-R. (2010*b*). *Cryst. Growth Des.* **10**, 3721–3726.

[bb7] Du, Z.-Y., Xu, H.-B. & Mao, J.-G. (2006). *Inorg. Chem.* **45**, 9780–9788. 10.1021/ic0613255 17112275

[bb8] Liang, J. & Shimizu, G. K. H. (2007). *Inorg. Chem.* **46**, 10449–10451. 10.1021/ic701628f 17997549

[bb9] Maeda, K. (2004). *Microporous Mesoporous Mater.* **73**, 47–55.

[bb10] Mao, J.-G. (2007). *Coord. Chem. Rev.* **251**, 1493–1520.

[bb11] Mao, J.-G., Wang, Z. K. & Clearfield, A. (2002). *Inorg. Chem.* **41**, 6106–6111. 10.1021/ic020396a 12425639

[bb12] Sheldrick, G. M. (2008). *Acta Cryst.* A**64**, 112–122. 10.1107/S0108767307043930 18156677

[bb13] Shimizu, G. K., Vaidhyanathan, H. R. & Taylor, J. M. (2009). *Chem. Soc. Rev.* **38**, 1430–1449. 10.1039/b802423p 19384446

[bb14] Song, J.-L. & Mao, J.-G. (2005). *J. Solid State Chem.* **178**, 3530–3537.

[bb15] Yang, B.-P., Prosvirin, A. V., Guo, Y.-Q. & Mao, J.-G. (2008). *Inorg. Chem.* **47**, 1453–1459. 10.1021/ic701351x 18225859

